# GAN-enhanced digital twins for biomarker-guided cancer immunotherapy and adoptive cell therapy: a mini review

**DOI:** 10.3389/fmed.2026.1894314

**Published:** 2026-09-29

**Authors:** Jiayu Yang

**Affiliations:** Shandong University of Technology, Zibo, Shandong, China

**Keywords:** adoptive cell therapy, cancer immunotherapy, digital twin, generative adversarial network, multimodal biomarkers, precision oncology, quantitative systems pharmacology

## Abstract

Cancer immunotherapy and adoptive cell therapy have transformed modern oncology, yet durable benefits remain limited by tumor heterogeneity, immune escape mechanisms, biomarker variability, and the dynamic remodeling of the tumor immune microenvironment. Current biomarker strategies, including PD-L1 (programmed death-ligand 1) expression, microsatellite instability (MSI), tumor mutational burden (TMB), immune gene signatures, liquid biopsy measurements, and target antigen profiles, provide clinically useful information but are often static and insufficient to capture longitudinal tumor–immune–therapy interactions. Digital twin technology offers a computational paradigm for integrating multi-omics, immune profiling, clinical variables, imaging, and treatment-response data into patient-linked virtual models. Generative artificial intelligence, including generative adversarial networks (GANs), may enrich sparse biomarker-defined state spaces and support the simulation of underrepresented treatment response or toxicity trajectories. This mini review critically compares mechanistic quantitative systems pharmacology, spatial and agent-based modeling, data-driven prediction, and emerging digital twin approaches and proposes a platform-agnostic architecture that can be implemented in MATLAB/Simulink or other computational environments. The adoptive cell therapy scope focuses on chimeric antigen receptor T-cell (CAR-T), chimeric antigen receptor natural killer cell (CAR-NK), and T-cell receptor-engineered T-cell (TCR-T) therapies, with related principles applicable to tumor-infiltrating lymphocyte (TIL) therapy. We describe conditional and longitudinal GAN strategies, dynamic tumor–immune state modeling, biomarker integration, validation metrics, governance, and prospective implementation pathways. The proposed framework is conceptual and has not yet been clinically validated; synthetic data are treated as methodological support rather than clinical evidence. Its intended near-term uses are hypothesis generation, virtual-cohort enrichment, trial design, and uncertainty-aware decision support.

## 1 Introduction

Cancer immunotherapy and adoptive cell therapy have generated durable responses in several malignancies; however, the clinical benefit remains uneven across tumor types and patient populations. Immune checkpoint inhibitors (ICIs), chimeric antigen receptor T-cell therapy, T-cell receptor-engineered therapy, and natural killer cell-based therapies are influenced by tumor antigenicity, antigen presentation, immune infiltration, effector cell fitness, immune escape, suppressive microenvironmental programs, and treatment-associated toxicity (1–3). This heterogeneity has made biomarker-driven decision-making central to precision immuno-oncology. In this review, adoptive cell therapy refers primarily to CAR-T, CAR-NK, and T-cell receptor-engineered T-cell (TCR-T) therapies; tumor-infiltrating lymphocyte (TIL) therapy is considered a related platform when the same biomarker and dynamic modeling principles apply.

Clinically approved or widely investigated biomarkers, such as PD-L1 expression, microsatellite instability, tumor mutational burden, circulating immune parameters, immune gene-expression signatures, and target antigen expression, provide useful but incomplete information (1). Many biomarkers are measured at a single time point, whereas tumor–immune interactions are dynamic. A baseline biopsy may not show treatment-induced antigen loss, immune editing, effector cell exhaustion, cytokine amplification, stromal remodeling, or acquired resistance. This gap is particularly important for cell therapy because therapeutic efficacy depends not only on tumor target expression but also on the trafficking, expansion, persistence, exhaustion, and survival of engineered immune cells (4–7).

Digital twin technology has emerged as a computational strategy to represent a patient or biological system as a continuously updated virtual counterpart. In oncology, digital twins may combine mechanistic modeling, quantitative systems pharmacology (QSP), artificial intelligence, multi-omics, and clinical monitoring to simulate tumor progression and treatment response (8–15). Generative artificial intelligence, including generative adversarial networks (GANs), may further address sparse-data problems by generating synthetic patient state profiles that preserve selected statistical and biological properties of real patient cohorts (16, 17). This mini review critically synthesizes recent evidence and proposes a translational, platform-agnostic architecture for biomarker-guided cancer immunotherapy and adoptive cell therapy. MATLAB/Simulink is retained as an interpretable implementation option rather than as a required platform, and no original computational or clinical validation results are presented.

Existing literature spans QSP virtual patients, spatial and agent-based models, multimodal predictive artificial intelligence, and early oncology digital twin concepts, but these strands are often evaluated separately. Recent AI immunotherapy reviews also emphasize therapeutic formulation and combination design rather than longitudinal patient-state updating (18, 19). The specific contribution of this mini review is to distinguish a dynamically updated digital twin from static prediction, define a restricted and auditable role for synthetic data, map clinically relevant biomarker domains to model states, and outline staged validation for checkpoint blockade and engineered cell therapies.

## 2 Biomarker domains requiring dynamic integration

Biomarkers in immuno-oncology can be organized into functional domains. The first domain involves tumor antigenicity. In checkpoint blockade, neoantigen burden, tumor mutational burden, and mismatch repair deficiency may indicate immunogenic potential, although predictive performance varies across tumor types (20–23). In cell therapy, target antigen expression is even more direct. CD19 (cluster of differentiation 19) and B-cell maturation antigen (BCMA) have supported major progress in hematological malignancies, whereas HER2 (human epidermal growth factor receptor 2), EGFR (epidermal growth factor receptor), mesothelin, CLDN18.2 (claudin 18.2), MUC1 (mucin 1), GD2 (disialoganglioside 2), and viral antigens, such as HPV (human papillomavirus) E6/E7, are being explored in solid tumors (4, 5, 7). Antigen heterogeneity and antigen loss, however, remain major mechanisms of resistance.

The second domain involves antigen presentation and interferon signaling. HLA (human leukocyte antigen) expression, B2M (beta-2-microglobulin) integrity, TAP1/TAP2 function, and interferon response programs influence whether tumor antigens can be effectively presented to T cells (24, 25). Defects in these pathways can produce primary or acquired resistance to checkpoint blockade and may influence TCR-engineered therapies. The third domain involves effector immune activity, including CD8A (CD8 alpha), GZMB (granzyme B), PRF1 (perforin 1), NKG7 (natural killer cell granule protein 7), GNLY (granulysin), and IFNG (interferon gamma)-associated cytotoxicity signatures. These markers capture immune attack more directly than tumor-intrinsic variables alone (1, 24).

The fourth domain involves checkpoint activation and immune exhaustion. PDCD1 (programmed cell death 1), CD274 (cluster of differentiation 274), CTLA4 (cytotoxic T-lymphocyte-associated protein 4), LAG3 (lymphocyte-activation gene 3), TIGIT (T-cell immunoreceptor with Ig and ITIM domains), HAVCR2 (hepatitis A virus cellular receptor 2), TOX (thymocyte selection-associated high mobility group box), and related exhaustion programs can indicate both an inflamed tumor state and functional impairment of effector lymphocytes (26–28). This dual interpretation is clinically important: Checkpoint expression may identify tumors susceptible to immunotherapy, but deep exhaustion, suppressive cytokines, and poor metabolic conditions may limit durable response. The fifth domain involves the immunosuppressive tumor microenvironment (TME). Regulatory T cells, myeloid-derived suppressor cells, tumor-associated macrophages, cancer-associated fibroblasts, TGF-β (transforming growth factor) signaling, hypoxia, adenosine metabolism, and abnormal vasculature may restrict immune infiltration and effector function (3, 24, 29, 30). Single-cell and spatial transcriptomic technologies increasingly show that response or resistance depends not only on the presence of immune cells but also on their spatial organization, functional states, and interactions with malignant and stromal compartments (29–32). These biomarker layers are interdependent and dynamic, which makes them suitable inputs for digital twin modeling rather than isolated static predictors.

Additional longitudinal biomarker domains include circulating tumor DNA (ctDNA) kinetics, circulating tumor cells (CTCs), T-cell receptor repertoire features, microbiome-associated signals, and metabolic fingerprints. Circulating genomic profiles may capture resistance and subsequent immunotherapy response, while recent multimodal and metabolic studies illustrate how imaging and metabolomics could broaden the observation layer of future digital twins (33–36). These studies are enabling examples rather than evidence that the corresponding data streams are already validated for digital twin-guided immunotherapy.

Clinical feasibility requires a tiered input strategy. A minimum layer may include tumor burden, treatment history, routine laboratory measurements, target antigen or HLA status, and selected liquid biopsy variables. Single-cell, spatial, microbiome, and metabolomic assays should remain optional research layers until their incremental value, reproducibility, and turnaround time are proven. Integration also requires time-stamped provenance, assay-specific quality control, unit harmonization, batch correction, explicit alignment to treatment events, and models that account for informative missingness. Platform-specific measurement uncertainty should propagate into the digital twin rather than being removed by preprocessing alone.

## 3 Digital twin modeling in immuno-oncology

A cancer digital twin is a patient-linked computational system that integrates biological data, clinical observations, and treatment inputs to simulate disease evolution and is updated when new measurements become available (10–15 56, 57). This definition differs from related terms. A virtual patient is a parameterized *in silico* individual sampled from a mechanistic model; a synthetic patient is a generated data record intended to resemble a source distribution; and a predictive model may provide a static or episodic estimate without bidirectional longitudinal updating. Maintaining these distinctions prevents synthetic cohorts or one-time risk models from being mislabeled as digital twins.

Current computational approaches can be grouped into mechanistic QSP or ordinary differential equation models, spatial or agent-based models, data-driven multimodal predictors, and hybrid digital twin architectures. QSP models provide interpretable links among pathway biology, dosing, immune cell dynamics, and response, but parameter identifiability and simplified biological assumptions limit personalization (11, 37–40). Spatial and agent-based models capture trafficking, tumor architecture, and local suppression but are computationally intensive and difficult to calibrate with routine patient data (31, 38, 39, 41). Data-driven models can integrate high-dimensional variables and achieve strong discrimination; however, they are often static, vulnerable to dataset shift, and less suitable for counterfactual schedule simulation (21–23, 33–36, 42, 43). Hybrid digital twins seek to combine these strengths through longitudinal calibration, but the majority of oncology implementations remain proof-of-concept, retrospectively validated, or aspirational (10–15, 44).

For biomarker-guided immunotherapy, a digital twin should include interacting representations of tumor burden and growth kinetics; target antigen and antigen presentation status; endogenous and engineered effector cell dynamics; suppressive microenvironmental signals; and treatment inputs such as checkpoint blockade, CAR-T or CAR-NK infusion, TCR-T therapy, lymphodepletion, cytokine support, or combination treatment. CAR-T and CAR-NK models require target density, trafficking, expansion, persistence, exhaustion, and cytokine toxicity states. TCR-T models additionally require HLA genotype, epitope presentation, B2M/TAP (transporter associated with antigen processing) integrity, and clonotype fitness. Such models could compare schedules and combinations, but clinically significant outputs must be calibrated to longitudinal observations and reported with uncertainty.

The proposed architecture does not assume that GAN augmentation or a MATLAB/Simulink implementation creates a digital twin by itself. GANs are assigned a restricted role in rare-state enrichment and stress testing, whereas the patient-linked mechanistic or hybrid core remains anchored to real longitudinal measurements. This separation is essential to the novelty and safety of the framework.

## 4 GAN-enhanced patient state enrichment

A major obstacle in biomarker-driven immunotherapy modeling is the scarcity, imbalance, and inconsistent longitudinal depth of high-quality datasets. Cell therapy cohorts are commonly small, disease-specific, and heterogeneous in prior treatment exposure. Rare but clinically important states, including early relapse after CAR-T therapy, severe cytokine release syndrome, immune effector cell-associated neurotoxicity syndrome, and acquired checkpoint-inhibitor resistance, are therefore underrepresented (7–9, 45, 46).

Candidate generative approaches include conditional GANs for outcome- or treatment-conditioned biomarker profiles, tabular GANs for mixed clinical and molecular variables, longitudinal adversarial models for serial trajectories, Wasserstein formulations with gradient penalties to improve training stability, and privacy-preserving variants for restricted data sharing (16, 17, 47–52). Conditioning variables include tumor type, treatment class, response state, antigen status, immune phenotype, time from infusion, and toxicity grade. Potential outputs include baseline multimodal profiles, serial biomarker trajectories, virtual-cohort parameter distributions, and rare response or toxicity states.

Synthetic data should not be interpreted as replacement evidence or used to create an apparent increase in clinical sample size for inferential claims. Evaluation must cover statistical fidelity, downstream utility, biological plausibility, subgroup robustness, temporal coherence, and privacy. Appropriate tests include train-on-synthetic/test-on-real and train-on-real/test-on-synthetic analyses, maintenance of pathway and marker co-expression constraints, outlier review, the calibration of downstream predictions, membership-inference risk, and performance in independent real cohorts. Risks include mode collapse, memorization and privacy leakage, hallucinated biomarker combinations, reinforcement of source-cohort bias, and overconfident predictions under distribution shift. Generated records should be labeled, versioned, auditable, and rejected when augmentation worsens calibration or minority group performance.

## 5 Implementation options and a MATLAB/Simulink-compatible architecture

### 5.1 Platform rationale and modular architecture

MATLAB/Simulink is well suited to state-space modeling, ordinary differential equations, parameter estimation, feedback control, sensitivity analysis, modular visualization, and co-simulation. These features support transparent block-level representation of tumor growth, immune cell dynamics, treatment inputs, and measurement updates. However, the framework is not platform-dependent. Python-based environments provide open-source machine learning, probabilistic programming, workflow automation, and deployment ecosystems; specialized QSP tools support pharmacology-centered model development; and agent-based platforms may better represent spatial cell–cell interactions. MATLAB/Simulink can facilitate rapid prototyping and traceable model-based design, but proprietary licensing, accessibility, version control, and deployment costs may limit adoption. Table 1 compares the principal computational paradigms.

**Table 1:** Comparison of computational paradigms for the proposed architecture.

Computational paradigm	Principal strengths	Main limitations	Role in the proposed architecture
Mechanistic QSP/ODE (ordinary differential equation) models	Biological interpretability; dose and schedule simulation; and counterfactual scenario testing (11, 37–40)	Parameter identifiability; simplified biology; and demanding calibration	Core tumor–immune treatment dynamics
Spatial or agent-based models	Cell trafficking, spatial heterogeneity, local interactions, and tissue architecture (31, 38, 39, 41)	High computational cost; difficult patient-level parameterization	Optional spatial TME and trafficking module
Data-driven multimodal models	High-dimensional integration and predictive discrimination (21–23, 33–36, 42, 43)	Opacity, dataset shift, static outputs, and weak counterfactual validity	Biomarker abstraction and observation models
Hybrid digital twins	Longitudinal updating, scenario comparison, and uncertainty-aware personalization (10–15, 44)	Limited prospective evidence; interoperability and governance barriers	Patient-linked overarching architecture
GAN and other synthetic data methods	Rare-state enrichment, class balancing, and stress testing (16, 17, 47–52)	Mode collapse, bias amplification, privacy leakage, and no new clinical evidence	Restricted support for training and sensitivity analysis

A practical architecture can be organized into five layers (Figure 1A). The data layer ingests multi-omics, single-cell and spatial transcriptomic measurements, pathology, radiomics, liquid biopsy, immune phenotyping, routine laboratory data, and treatment outcomes. The biomarker abstraction layer converts high-dimensional inputs into interpretable modules: antigenicity, antigen presentation, cytotoxic immunity, checkpoint/exhaustion, immune suppression, engineered cell fitness, and toxicity risk (Table 2). The generative layer may enrich underrepresented states under predefined biological, privacy, and fairness constraints. The dynamic model layer combines mechanistic QSP or state-space equations with data-driven observation models. The output layer reports response trajectories, relapse and toxicity risk, candidate schedules, and uncertainty rather than deterministic treatment recommendations.

**Figure 1:**
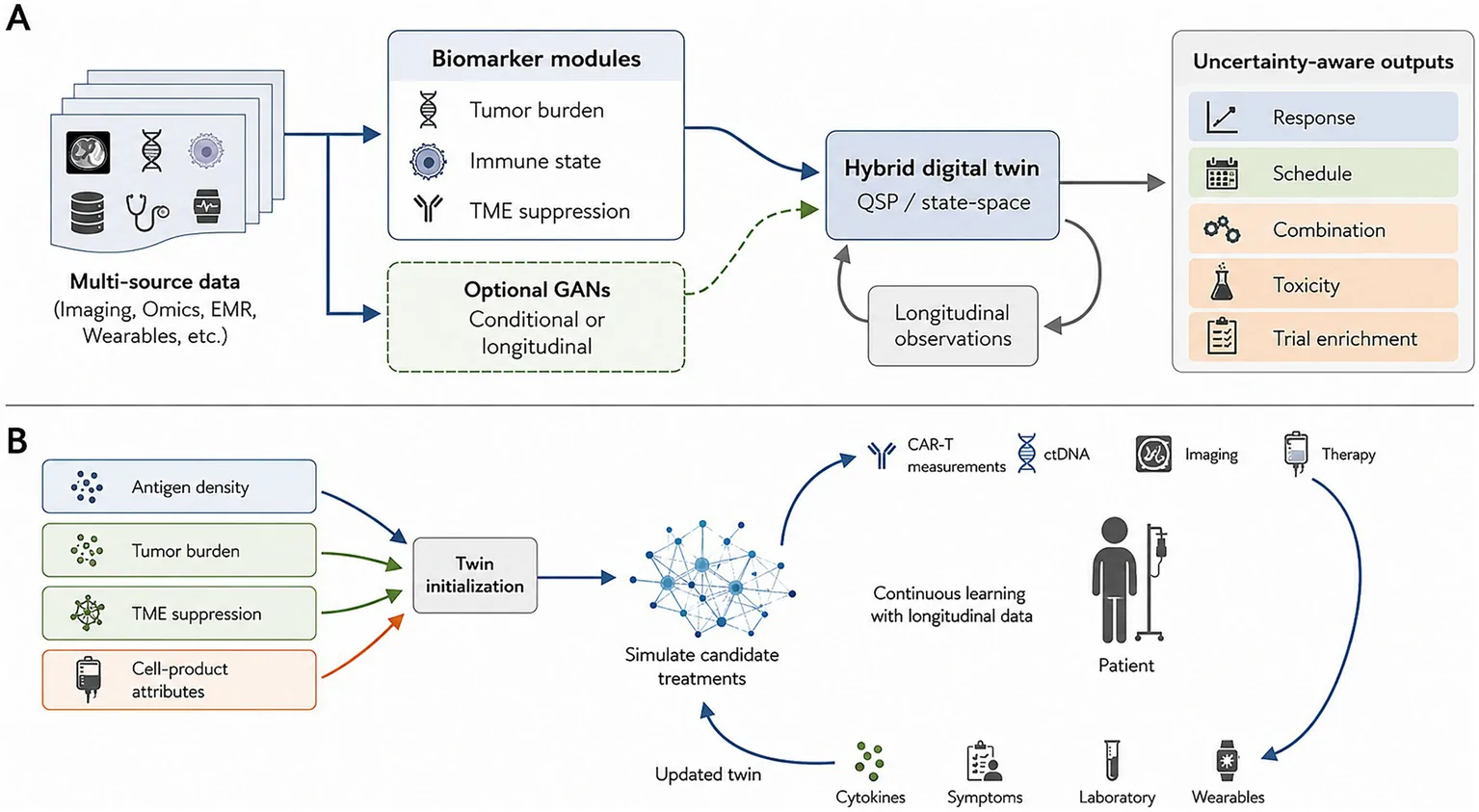
Two-panel framework for GAN-enhanced digital twins in biomarker-guided immunotherapy. **(A)** Multi-source data are transformed into interpretable biomarker modules, optionally enriched with conditional or longitudinal GANs under biological, privacy, and fairness constraints, and integrated into a hybrid QSP/state-space digital twin. The model is updated with longitudinal observations and produces uncertainty-aware predictions of treatment response, treatment schedules, combination strategies, toxicity, and trial-enrichment outputs. **(B)** Illustrative CAR-T application loop. Baseline antigen density, tumor burden, tumor microenvironment suppression, and cell product attributes initialize the twin; candidate treatment options are simulated; and CAR-cell measurements, ctDNA, imaging, cytokines, laboratory data, symptoms, and wearable signals update response, relapse, antigen loss, cytokine release syndrome (CRS), and immune effector cell-associated neurotoxicity syndrome (ICANS) estimates.

**Table 2:** Biomarker modules, digital twin roles, clinical applications, and representative evidence.

Biomarker module	Representative variables	Digital twin role	Potential clinical application
Tumor antigenicity	TMB, MSI status, neoantigen burden, CD19, BCMA, HER2, EGFR, MSLN (mesothelin), CLDN18.2, and HPV E6/E7 (1, 4, 7, 20–24)	Defines tumor immunogenicity and therapeutic targetability	ICI selection; CAR-T, CAR-NK, or TCR-T target prioritization
Antigen presentation	HLA, B2M, TAP1/TAP2, and interferon response genes (24, 25)	Regulates immune recognition and resistance risk	Immune escape assessment and TCR-T eligibility
Cytotoxic immune activity	CD8A, GZMB, PRF1, NKG7, GNLY, and IFNG (1, 24, 26–28)	Estimates effector pressure and killing potential	Response prediction and early on-treatment monitoring
Checkpoint/exhaustion	PDCD1, CD274, CTLA4, LAG3, TIGIT, HAVCR2, and TOX (1, 2, 26–28)	Models reversible and persistent immune dysfunction	ICI response prediction and combination design
Immunosuppressive TME	Treg, MDSC (myeloid-derived suppressor cell), TAM (tumor-associated macrophage), CAF (cancer-associated fibroblast), TGF-β, hypoxia, and adenosine metabolism (3, 24, 29–32)	Reduces infiltration, persistence, and killing efficiency	Selection of stromal, myeloid, or metabolic combinations
Engineered cell dynamics	CAR-T/CAR-NK expansion, persistence, trafficking, exhaustion; and TCR-T clonotype fitness (4–7, 53–55)	Simulates adoptive cell therapy kinetics	Dose, schedule, target, and combination optimization
Toxicity risk	C-reactive protein (CRP), ferritin, interleukin 6 (IL-6), cytokine signatures, tumor burden, fever, and neurologic findings (8, 9, 45, 46)	Represents CRS, ICANS, and immune-related adverse event risk	Risk-adapted monitoring and intervention planning
Longitudinal and emerging biomarkers	ctDNA, CTCs, TCR repertoire, microbiome features, metabolic fingerprints, and multimodal imaging (33–36, 42, 43)	Updates latent disease and immune states between tissue assessments	MRD (minimal residual disease)/relapse monitoring, resistance detection, and adaptive reassessment

In a simplified state-space formulation, *x*(*t*) = [*B*(*t*), *E*(*t*), *A*(*t*), *X*(*t*), *S*(*t*), *C*(*t*)] may represent tumor burden, endogenous effector activity, adoptive cell abundance, exhaustion, immunosuppressive pressure, and cytokine toxicity state. The dynamics can be written conceptually as *dx*/*dt* = f(*x*, *u*, theta, *z*) + epsilon, where *u*(*t*) encodes treatment inputs, theta contains patient-specific parameters, and *z* contains biomarker-derived covariates. Observations *y*(*k*) = *h*[*x*(*tk*)] + eta link latent states to imaging, ctDNA, immune cell counts, CAR transgene measurements, cytokines, and symptoms. Parameter estimation may combine non-linear optimization or Bayesian calibration with global sensitivity and identifiability analysis. Sequential observations can update latent states through ensemble Kalman or particle filter approaches, and outputs should be predictive distributions with calibrated intervals.

### 5.2 Illustrative CAR-T digital twin use case

Figure 1B provides a concrete application example. Baseline target antigen density, tumor burden, anatomic distribution, suppressive tumor microenvironment features, and cell product attributes initialize the model. The twin then compares candidate infusion timing, lymphodepletion, dose, and checkpoint- or TME-modulating combinations. During treatment, CAR transgene or circulating cell counts, ctDNA, imaging, cytokines, routine laboratory data, symptoms, and wearable measurements can recalibrate the latent states. The output is not a single prescription but a set of responses, antigen loss, relapse, cytokine release syndrome, and neurotoxicity probabilities with explicit uncertainty. Equivalent logic can be applied to CAR-NK therapy, whereas TCR-T models require HLA-restricted epitope presentation and antigen-processing integrity as additional constraints (4–8, 24, 25, 45, 46, 53–55).

## 6 Translational applications

The first application is patient stratification for immune checkpoint blockade. A biomarker-guided digital twin could integrate tumor-intrinsic biomarkers, immune infiltration, blood-based parameters, radiomic features, and spatial immune organization to move beyond single-marker prediction (1, 21–24, 29–31, 42, 43). Instead of classifying patients only at baseline, the model could simulate early on-treatment changes and identify when adaptive treatment modification may be needed. Dynamic inputs may include ctDNA kinetics, serial blood immune phenotyping, T-cell receptor repertoire shifts, and early imaging changes, but their incremental value should be assessed against a parsimonious clinical baseline rather than assumed.

The second application involves target and patient selection for engineered cell therapy. For CAR-T and CAR-NK therapy, a digital twin can combine surface antigen density and heterogeneity, trafficking barriers, expansion and persistence, prior therapy, suppressive myeloid or stromal states, and cytokine toxicity risk (4–7, 53–55). For TCR-T therapy, the model must additionally represent HLA restriction, intracellular epitope abundance, antigen processing, clonotype avidity, and potential off-target recognition. TIL therapy may use related modules focused on clonotype composition, *ex vivo* fitness, tumor reactivity, and reinfusion kinetics. These platform-specific constraints are essential because the same biomarker cannot be interpreted identically across CAR-, TCR-, NK-, and TIL-based therapies.

The third application is combination therapy optimization. Checkpoint blockade, cytokine modulation, targeted therapy, radiotherapy, cancer vaccines, nanomedicine, and stromal-modulating approaches may be combined with cell therapy (3, 5, 7, 18, 19). A calibrated model could distinguish whether a candidate combination primarily improves antigen release, effector expansion, infiltration, exhaustion reversal, or suppression reduction. The fourth application is toxicity monitoring. Cytokine release syndrome, neurotoxicity, and immune-related adverse events require models that integrate baseline inflammation, tumor burden, immune cell expansion kinetics, cytokines, organ function, and early clinical measurements (8, 9, 45, 46). Safety outputs require conservative thresholds, prospective validation, and explicit abstention when data are outside the model’s validated domain.

Recent AI-enabled immunotherapy research also includes molecular delivery and combination design applications, such as AI-guided liposomal nanocarriers and AI-assisted chemo-immunotherapy synthesis (18, 19). These approaches optimize therapeutic components, whereas a digital twin models the evolving state of an individual patient. The two levels may become complementary when AI-generated regimen candidates are evaluated within calibrated virtual cohorts and subsequently tested in prospective studies.

## 7 Validation, governance, limitations, and future directions

### 7.1 Data integration, integrity, and feasibility

Clinical implementation requires a common data model that records specimen type and site, assay method, processing batch, acquisition time, treatment exposure, missingness, and measurement uncertainty. Batch correction cannot substitute for prospective harmonization, and measurements obtained at different disease stages should not be treated as interchangeable. A tiered workflow should distinguish routinely available variables from optional research assays so that model performance can be evaluated under realistic resource constraints.

Generative augmentation adds separate limitations. Mode collapse, privacy leakage, subgroup distortion, unrealistic biomarker combinations, and apparent performance gains caused by data leakage can invalidate all downstream conclusions (16, 17, 47–49, 51). Mechanistic models also face structural and practical identifiability problems: Multiple parameter sets may reproduce the same trajectory, and increased model complexity can reduce rather than improve clinical reliability. These limitations require prespecified model simplification, sensitivity analysis, negative controls, and documentation of unsupported states.

### 7.2 Staged validation and performance metrics

Validation should progress through retrospective development, temporal validation, geographic external validation, prospective silent deployment, and, finally, interventional clinical utility testing. Data partitioning should occur at the patient and, where possible, institutional levels. External validation should include different cancer types, treatment protocols, assay platforms, demographic groups, and prevalence of rare toxicities. A model should be recalibrated rather than simply transported when baseline risk or measurement processes differ.

Performance assessment should match the intended output. Binary predictions require discrimination and precision–recall metrics, calibration slope and intercept, Brier score, decision curve analysis, and subgroup evaluation. Longitudinal trajectories require time-resolved error, predictive interval coverage, and stability after sequential updates. Survival outputs require time-dependent concordance and calibration. Synthetic data evaluation should include downstream performance on untouched real cohorts, privacy attacks, fairness, and sensitivity of the digital twin output to generated samples. Prospective studies should predefine acceptable error, uncertainty, and abstention thresholds.

### 7.3 Governance and clinical implementation

Clinical use requires a predefined intended use, transparent human oversight, version control, audit logs, data provenance, model change management, and clear responsibility for decisions. Patient consent should address longitudinal data linkage and potential synthetic data generation. Outputs should communicate confidence intervals, data quality warnings, and out-of-distribution status. A digital twin should support, not replace, multidisciplinary judgment, and it should not automatically recommend treatment when evidence is incomplete or uncertainty is high.

### 7.4 Future research priorities

Near-term priorities are prospective multi-institutional cohorts with standardized longitudinal biomarker panels, interoperable data infrastructure, reproducible benchmark tasks, and transparent reporting of calibration and uncertainty. A practical development sequence is prospective silent validation, followed by biomarker-enriched or window-of-opportunity studies, adaptive trials that predefine model updates, and randomized evaluation of digital twin-assisted decision support and standard planning.

Real-time monitoring may incorporate ctDNA, peripheral immune phenotyping, CAR transgene or cell counts, cytokines, routine laboratory data, imaging, symptoms, and wearable vital signs. These streams can trigger model updates, but not automatic treatment changes. Even with improved data, unresolved barriers include causal validity, parameter identifiability, clinical workflow burden, software licensing and accessibility, computational requirements, and the risk that underrepresented populations receive less reliable predictions.

## 8 Conclusion

GAN-enhanced digital twins are a promising but still conceptual approach to biomarker-guided cancer immunotherapy and adoptive cell therapy. Their value depends on a disciplined separation of synthetic data generation, mechanistic or hybrid modeling, longitudinal patient updates, and independent clinical validation. A platform-agnostic modular architecture can support research on checkpoint blockade, CAR-T, CAR-NK, TCR-T, TIL, combination therapy, and toxicity, but its near-term role is virtual-cohort enrichment, trial design, and uncertainty-aware decision support rather than autonomous treatment prescription.
